# Dose escalation of radiotherapy in unresectable extrahepatic cholangiocarcinoma

**DOI:** 10.1002/cam4.1734

**Published:** 2018-08-27

**Authors:** Dalia Elganainy, Emma B. Holliday, Cullen M. Taniguchi, Grace L. Smith, Rachna Shroff, Milind Javle, Kanwal Raghav, Ahmed Kaseb, Thomas A. Aloia, Jean Nicolas Vauthey, Ching‐Wei D. Tzeng, Joseph M. Herman, Albert C. Koong, Sunil X. Krishnan, Bruce D. Minsky, Christopher H. Crane, Prajnan Das, Eugene J. Koay

**Affiliations:** ^1^ Department of Radiation Oncology UT MD Anderson Cancer Center Houston Texas; ^2^ Department of GI Medical Oncology UT MD Anderson Cancer Center Houston Texas; ^3^ Department of Surgical Oncology UT MD Anderson Cancer Center Houston Texas; ^4^ Department of Radiation Oncology Memorial Sloan Kettering Cancer Center New York New York

**Keywords:** dose escalation, extrahepatic cholangiocarcinoma, radiation therapy, toxicity, unresectable extrahepatic cholangiocarcinoma

## Abstract

**Purpose:**

To evaluate the effect of escalated dose radiation therapy (EDR, defined as doses >50.4 Gy in 28 fractions [59.5 Gy BED]) on overall survival (OS), freedom from local progression (FFLP), and freedom from distant progression (FFDP) of patients with unresectable extrahepatic cholangiocarcinoma (EHCC).

**Methods:**

A consecutive cohort of 80 patients who underwent radiotherapy for unresectable EHCC from 2001 to 2015 was identified. Demographic, tumor, treatment, toxicity, and laboratory variables were collected. The maximal RT doses ranged from 30 to 75 Gy (median 50.4 Gy, at 1.8‐4.5 Gy/fraction). Gross tumor volume (GTV) coverage by maximal dose in EDR group ranged from 38% to 100%. Kaplan–Meier method was used to estimate OS, FFLP, and FFDP. Univariate and multivariate Cox regression models were analyzed.

**Results:**

After radiotherapy, median OS, FFLP, and FFDP were 18.7, 22.6, and 24.3 months, respectively. There was no significant difference in OS or FFLP between patients who received EDR to portions of the GTV and patients who did not. On multivariate analysis, bigger GTV, age, and ECOG performance status were independently associated with shorter OS. Local progression on chemotherapy prior to RT was independently associated with shorter FFLP. High baseline neutrophil/lymphocyte ratio (>5.3) was independently associated with shorter FFDP. Toxicity grades were similar in EDR and lower doses except lymphopenia which was higher in EDR (*P* = 0.053).

**Conclusions:**

EDR to selective portions of the GTV may not benefit patients with unresectable EHCC despite having acceptable toxicity. New methods to improve local control and survival for unresectable EHCC are needed.

## 1 INTRODUCTION

Extrahepatic cholangiocarcinoma (EHCC) is a rare and lethal malignancy that originates from the epithelial cells of the extrahepatic bile ducts. EHCC can be further divided according to its location into perihilar and distal types. According to a Surveillance, Epidemiology, and End Results (SEER) review from 1975 to 2013, the incidence is 1.9 cases per 100 000 people,^1^ and the incidence appears to be increasing. Overall, the prognosis of EHCC is poor, with a 5‐year survival rate of 16.9%.^1^ Currently, complete surgical resection is the only potentially curative treatment for anatomically resectable tumors, but most patients present with unresectable locally advanced or metastatic disease due to late presentation and nonspecific symptoms.^2^, ^3^, ^4^ Resectability is determined by local extent of the tumor including vascular involvement, estimated magnitude of pancreatic or liver resection, and metastatic disease.^5^ Effective treatment options for these patients are needed.

The Southwest Oncology Group 0809 study demonstrated an encouraging median survival of 35 months for 79 patients with resected EHCC (68% of patients) or gallbladder cancer (32% of patients) who received adjuvant capecitabine/gemcitabine followed by chemoradiation.^6^ The role of adjuvant capecitabine has also been recently investigated in the BILCAP study.^7^ In patients with locally advanced disease, the data are sparse. In unresectable nonmetastatic EHCC, radiation therapy (RT) with or without concurrent chemotherapy has been the treatment of choice as it has an important role in controlling local disease progression,^8^ which is a major cause of treatment failure in these patients.^9^ Studies have also reported improved survival with radiation treatment in unresectable EHCC with different intents.^10^, ^11^, ^12^, ^13^ In a series of 52 patients with locally advanced EHCC treated at our institution, we previously identified the limitations of conventional doses of radiotherapy in this disease and suggested that a possible way to overcome that would be escalated dose radiotherapy (EDR).^14^ After that analysis, we have selectively escalated the radiation dose to levels that would be considered definitive in other solid tumors while incorporating new technologies such as intensity‐modulated RT, image guidance, and respiratory motion control as they emerged.

Given the rarity of this malignancy, evidence that definitive doses of RT lead to long‐term survival in these patients is lacking, and a clear dose‐response relationship has not been established.

Within this context, we reviewed our experience with patients with unresectable EHCC who were treated with radiotherapy with or without concurrent chemotherapy, looking for evidence of long‐term local tumor control, and overall survival. Exploratory analysis was also carried out to identify any other clinical factors that were predictive of clinical outcome.

## 2 PATIENTS AND METHODS

### 2.1 Patients

After institutional review board approval of this retrospective study (PA14‐0646), we identified a consecutive series of 89 patients with unresectable EHCC who received radiotherapy at The University of Texas MD Anderson Cancer Center from 2001 to 2015. Of these, we excluded nine patients who were diagnosed with metastatic disease before treatment. Disease was identified as unresectable based on radiographic or intraoperative findings of main portal vein involvement, nodal metastasis, involvement of secondary biliary radicals, insufficient liver remnant volume, or medical inoperability. Most patients (61 of 80) had their disease confirmed with pathologic examination, all other patients (19 of 80) were diagnosed based on classic presentation on radiologic imaging or endoscopic retrograde cholangiopancreatography. Sixty‐two patients (77.5%) had perihilar EHCC and eighteen patients (22.5%) had distal EHCC. Staging was according to American Joint Committee on Cancer 7th edition.^15^

### 2.2 Treatment

**Figure 1:**
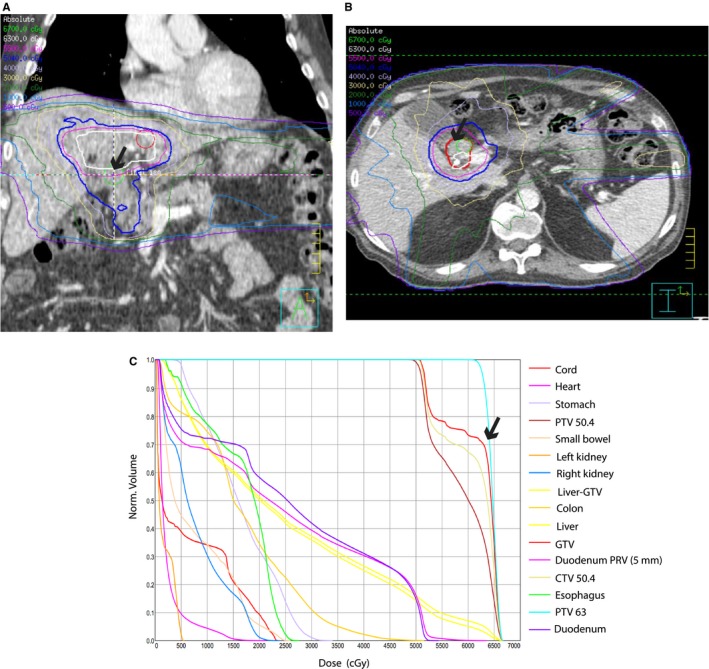
A and B, Images from radiation therapy plan of a patient who was prescribed 225 cGy per fraction for 28 fractions. 70% of gross tumor volume (GTV, thick red contour) was covered by the maximal dose (63 Gy, white contour). Arrows show areas of tumor not covered with maximal dose. C, Dose‐volume histogram (DVH) showing the percentage of GTV that was covered by maximal prescribed dose

At our institution, we have routinely treated locally advanced EHCC with external beam RT usually if there is lack of metastatic progression after at least several months of systemic chemotherapy. RT was delivered by 3D conformal technique, intensity‐modulated radiation therapy (IMRT), or passive scatter proton beam technique. EDR (defined as delivery of higher than 50.4 Gy in 28 fractions [biological equivalent dose (BED) >59.5 Gy assuming α/β of 10 Gy for tumor^16^]) was delivered in patients using a combination of technologies that enabled higher doses to portions of the gross tumor volume (GTV) that was away from bowel (Figure 1). The technologies included conformal RT with IMRT, daily image guidance, and respiratory motion management. Breath‐hold technique was used in 13 patients, and CT on rails or cone beam CT image guidance was used in 16 patients. Even with the use of these technologies, bowel dose constraints limited the coverage of the GTV in the EDR group (maximal RT dose coverage ranged from 38% to 100% of the GTV). Based on physician preference, most patients received concurrent chemotherapy with radiotherapy (n = 69). The majority were capecitabine‐based (62 of 69). Two of these patients had their capecitabine held during the first 2 weeks due to abdominal pain and nausea in one patient and clostridium difficile enterocolitis in the other. Other patients received 5‐fluorouracil (n = 6) or gemcitabine (n = 1). Twenty‐three patients received induction chemotherapy before RT, and 34 patients received chemotherapy after RT. None of the patients underwent oncologic resection. Treatment characteristics are detailed in Table 1. Patients who developed ascites for obvious clinical reasons other than RT effect were not considered in the late toxicity analysis. Acute toxicity was evaluated according to the Common Terminology Criteria for adverse events v4.03.^17^

**Table 1:** Patient and treatment characteristics for all patients and by type of EHCC

Baseline patient characteristics (n=80)	All patients	Perihilar Cholangiocarcinoma (Klatskin's tumor)	Distal Cholangiocarcinoma
Number [%]	80	62 [77.5]	18 [22.5]
Age in years			
Mean	66.8	66.85	66.77
Median [range]	68.5 [30‐87]	68.5 [30‐86]	68 [51‐87]
Gender [%]			
Male	47 [59]	38 [61]	9 [50]
Female	33 [41]	24 [39]	9 [50]
Race [%]			
White	61 [76]	50 [81]	11 [61]
Other	14 [18]	9 [14]	5 [28]
Unknown	5 [6]	3 [5]	2 [11]
ECOG Scale of performance status [%]			
0	31 [39]	25 [40]	6 [33]
1	37 [46]	26 [42]	11 [61]
2	10 [13]	9 [15]	1 [6]
3	2 [2]	2 [3]	
Baseline CA 19‐9 level (n=71)			
Median [range]	174.6 [1‐16 050]	192.1 [1‐16 050]	28.9 [1‐313]
Portal vein involvement [%]	22 [28]	17 [27]	5 [28]
Bismuth‐Corletteclassification of perihilar EHCC [%]			
Type I		3 [5]	
Type II		1 [2]	
Type IIIA		12 [19]	
Type IIIB		12 [19]	
Type IV		34 [55]	
T classification			
1	1 [1]	1 [2]	
2	15 [19]	8 [13]	7 [39]
3	33 [41]	27 [44]	6 [33]
4	31 [39]	26 [42]	5 [28]
N classification			
0	38 [48]	27 [44]	11 [61]
1	41 [51]	34 [55]	7 [39]
2	1 [1]	1 [2]	0
Overall stage		Stage [n]	Stage [n]
		I [1]	IB [4]
		II [3]	IIA [4]
		IIIA [9]	IIB [2]
		IIIB [18]	III [5]
		IVA [23]	
		IVB [1]	
Median radiation dose (Gy) [range]	50.4 [30‐75]	54 [30‐75]	50.4 [50.4‐75]
Median BED [range]	59.5 [36‐98]	63 [36‐98]	59.5 [59.5‐98]
Radiation dose group N [%]			
BED >59.5	37 [46]	33 [53]	4 [22]
BED ≤59.5	43 [54]	29 [47]	14 [78]
BED >77	18 [22.5]	15 [24]	3 [17]
BED ≤77	62 [77.5]	47 [76]	15 [83]
Concurrent chemotherapy			
Yes	69 [86]	54 [87]	15 [83]
No	11 [14]	8 [13]	3 [17]
Radiation technique N [%]			
IMRT	44 [55]	38 [61]	6 [33]
Conventional 3D conformal	35 [44]	23 [37]	12 [67]
3D proton beam	1 [1]	1 [2]	0 [0]
RT fractionation regimens N [%]			
50.4Gy in 28 fractions	34 [41]	20 [32]	14 [78]
63Gy in 28 fractions	6 [8]	6 [10]	0 [0]
68.4Gy in 38 fractions	6 [8]	5 [8]	1 [6]
75Gy in 25 fractions	4 [5]	2 [3]	2 [11]
67.5Gy in 15 fractions	2 [2.5]	2 [3]	0 [0]
Other regimens	28 [36]	27 [44]	1 [6]
Year treated N [%]			
2001‐2009	38 [48]	31 [50]	7 [39]
2010‐2015	42 [52]	31 [50]	11 [61]

ECOG, Eastern Cooperative Oncology Group; CA 19‐9, carbohydrate antigen 19‐9; EHCC, extrahepatic cholangiocarcinoma; Gy, gray; BED, biological equivalent dose; RT, radiotherapy; IMRT, intensity‐modulated radiation therapy.

### 2.3 Statistical analysis

We statistically analyzed patient demographics, tumor variables, treatment variables, acute and late toxicity, freedom from local progression (FFLP), freedom from distant progression (FFDP), and overall survival (OS). Causes of death were also analyzed. Statistical analysis was performed using JMP pro 12 (SAS, North Carolina, USA). The Kaplan–Meier method was used to estimate FFLP, FFDP, and OS, and compared using log‐rank test with 95% confidence intervals. FFLP was defined as the time between date of RT start and date of radiological local progression or first sign of clinical local progression. FFDP was defined as the time between date of RT start and date of first radiological evidence of hepatic or extrahepatic metastasis. OS was defined as the time between start of radiotherapy date and the date of death or last follow‐up. Differences in event rates between groups were calculated using log‐rank test. The association of each variable with OS, FFLP, and FFDP was derived from a Cox proportional hazards model. All tests were two‐sided, and *P* value ≤0.05 was considered statistically significant.

## 3 RESULTS

A total of eighty patients received RT for nonmetastatic unresectable EHCC during the period between 2001 and 2015. There were no significant differences in patient demographics between patients with perihilar cholangiocarcinoma and those with distal cholangiocarcinoma. Median age for all patients was 68.5 years (range, 30‐87 years). 59% were male and 76% were Caucasian. Detailed patient characteristics in perihilar and distal cholangiocarcinoma are listed in Table 1.

### 3.1 Radiation dose and outcomes

**Figure 2:**
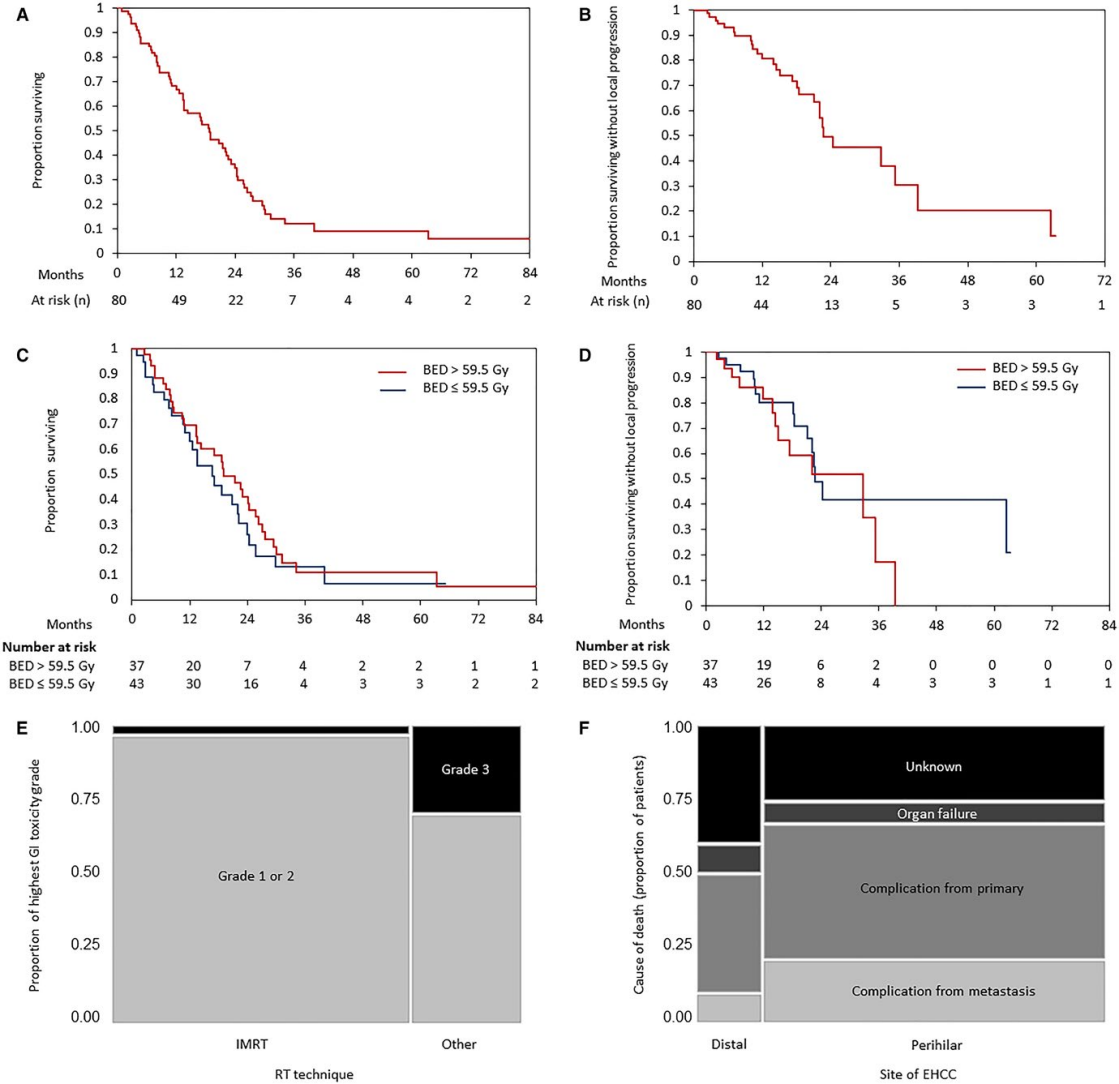
A, Overall survival and B, local control of all patients who received definitive radiation therapy for unresectable cholangiocarcinoma. Comparison of overall survival (C), freedom from local progression (D) between patients treated with biological equivalent dose (BED) >59.5 Gy vs BED ≤59.5 Gy. E, Contingency analysis of highest GI toxicity grades sorted by RT technique in patients who received EDR, and F, causes of death in perihilar and distal EHCC

Median RT dose was 50.4 Gy (range, 30‐75 Gy), and median treatment duration was 37 days (range, 13‐64 days). Median BED was 59.5. 37 patients received RT doses higher than 50.4 Gy. Doses of RT used from 2001 to 2015 in this cohort of patients are described in Figure S1. Characteristics of patients who received RT doses more than 50.4 Gy vs those who received doses less than or equal to 50.4 Gy are described in Table 2. EDR (>50.4 Gy) to portions of the GTV away from bowel did not significantly affect OS or FFLP in all patients (*P* = 0.4 and *P* = 0.4, respectively; Figure 2C,D). It also did not significantly affect OS or FFLP in either the perihilar or the distal EHCC cohorts. Other cutoffs of the radiation dose also did not significantly affect OS or FFLP (data not shown). Analysis revealed no significant differences in OS or FFLP between 3D conformal RT and IMRT. Also, there was no significant difference in OS or FFLP between patients who were treated with RT alone and patients who were treated with chemoradiation therapy (Table S1).

**Table 2:** Characteristics of patients by treatment with BED >59.5 Gy vs BED ≤59.5 Gy

Characteristic	Patients treated with BED >59.5 Gy N (% or range)	Patients treated with BED ≤59.5 Gy N (% or range)	*P* value
Number of patients	37	43	
Median age (y)	69 (30‐86)	68 (31‐87)	0.37*
Gender			0.82†
Male	21 (43)	26 (60)	
Female	16 (57)	17 (40)	
Race			0.84†
White	28 (76)	33 (77)	
Other	6 (16)	8 (18)	
Unknown	3 (8)	2 (5)	
ECOG scale of performance status			0.23†
0	11 (30)	20 (46)	
1	19 (51)	18 (42)	
2	5 (14)	5 (12)	
3	2 (5)	0	
Median baseline CA 19‐9	182.6 (8.5‐16 050)	136.8 (1‐3026)	0.12*
Median GTV (cm^3^)	46.5 (5‐360)	63 (6‐548)	0.18*
Overall stage			0.12†
I	1 (3)	5 (12)	
II	4 (11)	7 (16)	
III	15 (40)	21 (49)	
IV	17 (46)	10 (23)	
Portal vein involvement			1†
Yes	10 (27)	12 (28)	
No	27 (73)	31 (72)	
EHCC type			
Perihilar	33 (89)	29 (67)	0.03†
Distal	4 (11)	14 (33)	
RT fractionation regimens N [%]			
50.4 Gy in 28 fractions	0	34	
63 Gy in 28 fractions	6	0	
68.4 Gy in 38 fractions	6	0	
75 Gy in 25 fractions	4	0	
67.5 Gy in 15 fractions	2	0	
Other regimens	19	9	
Concurrent chemotherapy			0.74†
Yes	31 (84)	38 (88)	
No	6 (16)	5 (12)	
Local progression	13 (46)	15 (54)	1†

RT, radiotherapy; ECOG, Eastern Cooperative Oncology Group; GTV, gross tumor volume; EHCC, extrahepatic cholangiocarcinoma.

*Mann‐Whitney *U* test.

†Fisher's exact test.

### 3.2 Survival and patterns of failure

#### 3.2.1 Overall survival and cause of death

At the time of analysis, 19 patients (24%) were still under observation or lost to follow‐up. The Kaplan‐Meier estimate of mean overall survival was 21.3 months, and the median survival time was 18.7 months (Figure 2A). There was a significant difference in OS between patients with perihilar EHCC tumors (median OS = 16.7 months) and patients with distal EHCC tumors (median OS = 27.1 months) (log‐rank, *P* = 0.007). Predictors of OS in univariate survival analysis were baseline neutrophil/lymphocyte ratio (NLR as a continuous variable, *P* = 0.04), normalized baseline CA19‐9 (as a continuous variable, *P* = 0.0007), local progression on chemotherapy prior to RT (*P* = 0.02), portal vein involvement (*P* = 0.04), GTV (*P* = 0.002), overall stage (*P* = 0.002), and ECOG performance status (*P* = 0.008; Table S1). In multivariate survival analysis, higher GTV, age, and ECOG performance status were independently associated with shorter OS (Table 3). We classified long‐term and short‐term survivors with a cutoff of 36 months, and we then defined NLR cutoff of 5.3 using receiver operating characteristics curve analysis. OS was significantly longer in patients with NLR ≤5.3 in comparison with patients with NLR >5.3 in univariate analysis (log‐rank test, *P* = 0.002, Figure S3A). Of the 61 patients who died, 27 died of biliary or liver complications from the primary tumor. This included cholangitis (n = 7), biliary obstruction (n = 6), liver failure (n = 3), portal vein occlusion (n = 2), and combined causes of two or more of the previous (n = 9). Twelve patients died from complications of metastatic disease. Five patients died of organ failure which included renal failure (n = 3), cardiac failure (n = 1), and multi‐organ failure (n = 1). Seventeen patients had an unknown cause of death (Figure 2F).

**Table 3:** Multivariate overall (OS), freedom from local progression (FFLP), and freedom from distant failure (FFDP) survival analyses

	Definition	Multivariate OS analysis		Multivariate FFLP analysis		Multivariate FFDP analysis	
		*P* value	HR [95% CI]	*P* value	HR [95% CI]	*P* value	HR [95% CI]
Baseline NLR^a^		0.6	1.026 [0.9‐1.11]	0.07	1.15 [1.01‐1.31]	0.09	1.1 [0.98‐1.24]
Site of EHCC	Perihilar	0.7	1.22 [0.44‐3.63]			0.3	1.7 [0.58‐5.8]
	Distal	Reference				Reference	
Local progression on chemotherapy prior to RT	Yes	0.1	2.29 [0.82‐5.56]	**0.01**	5.6 [1.5‐17.7]	0.2	2.4 [0.57‐7.8]
	No	Reference		Reference		Reference	
Portal vein involvement	Yes	0.08	1.82 [0.93‐3.5]	0.2	1.7 [0.66‐4.3]		
	No	Reference		Reference			
GTV^a^		**0.01**	1.004 [1‐1.007]			0.5	1.002 [0.99‐1.006]
Age^a^		**0.004**	1.04 [1.01‐1.07]				
Gender	Male			0.3	0.7 [0.29‐1.45]		
	Female			Reference			
Overall stage	III or IV	0.2	2.2 [0.74‐7.34]	0.6	1.3 [0.48‐4.2]		
	I or II	Reference		Reference			
ECOG performance status	2 or 3	**0.04**	2.96 [1.07‐7.33]			0.9	0.99 [0.19‐3.8]
	0 or 1	Reference				Reference	
Radiation dose	High (>50.4 Gy)					0.7	1.19 [0.5‐2.9]
	Standard or low (≤50.4 Gy)					Reference	
Use of concurrent chemotherapy	Yes					0.3	0.5 [0.19‐1.9]
	No					Reference	

HR, hazards ratio; CI, confidence interval; NLR, neutrophil lymphocyte ratio; RT, radiotherapy; EHCC, extrahepatic cholangiocarcinoma; ECOG, Eastern Cooperative Oncology Group.

a As a continuous variable.

*P* values in bold indicate statistical significance.

#### 3.2.2 Local progression

Local progression was diagnosed in 28 patients after the completion of RT. Median time of FFLP was 22.6 months (Figure 2B). Local progression on chemotherapy prior to RT was associated with worse FFLP in univariate analysis (Table S1). Local progression on chemotherapy prior to RT was independently associated with worse FFLP in multivariate analysis (Table 3). The main sites of local progression were the liver hilum (n = 11) in perihilar EHCC and the common bile duct (n = 4) in distal EHCC. FFLP was also significantly longer in patients with NLR ≤5.3 in comparison with patients with NLR >5.3 in univariate analysis (log‐rank, *P* = 0.006, Figure S3B) and in multivariate analysis (Table S2). There was no significant difference in FFLP between IMRT and other techniques (log‐rank, *P* = 0.8).

#### 3.2.3 Distant progression

Overall, 32 patients developed distant metastasis in one or more sites after RT. The main sites of distant metastasis were the peritoneum (n = 15), the liver (n = 13), and the lung (n = 13). Median time of FFDP was 24.3 months (Figure S2). Higher baseline NLR, perihilar site of EHCC, not using concurrent chemotherapy, and radiation dose >50.4 Gy were associated with worse FFDP in univariate analysis (Table S1). Baseline NLR demonstrated a trend of being independently associated with FFDP as a continuous variable (Table 3). FFDP was significantly longer in patients with NLR ≤5.3 in comparison with patients with NLR >5.3 in univariate analysis (log‐rank, *P* = 0.0005, Figure S3C) and multivariate analysis (Table S2).

### 3.3 Toxicity

RT alone and chemoradiation therapy (CRT) were generally well tolerated. The rate of severe acute gastrointestinal (GI) toxicity (Grade 3+) was 11% in all patients. In RT doses higher than 50.4 Gy, grade 3+ GI toxicities were 3.7% for IMRT (1/27 patients), while for the other two techniques used they were 33% (3/10 patients, *P* = 0.03) (Figure 2E). The rate of severe (Grade 3+) acute other toxicities (excluding hematological toxicities) was 15% in all patients. Grades of most common acute toxicities during RT/CRT and late effects are listed in Table 4 for all patients and by dose of RT. In general, higher RT doses were not associated with higher grade toxicities (excluding hematological toxicities) (*P* = 0.8). However, grade 3+ lymphopenia during treatment was correlated with RT doses higher than 50.4 Gy (Fisher's exact, *P* = 0.053). This could also be chemotherapy‐related toxicity as most of the patients received concurrent chemotherapy. Twenty‐six patients were hospitalized within 90 days of RT completion, of whom 10 were hospitalized due to therapy‐related biliary complications (mainly cholangitis) and six were hospitalized due to GI bleeding. 28% late toxicity was recorded consisting of ascites (30 patients) and GI bleeding (11 patients). 50% of patients who developed ascites required management.

**Table 4:** Grades of most common acute toxicities and late effects during RT^b^ for all patients and by treatment with biological equivalent dose (BED) >59.5 Gy vs BED ≤59.5 Gy

Toxicity type and grade (total number)	All patients N [% from n of toxicity]	Patients treated with BED > 59.5 Gy N [% from n of grade]	Patients treated with BED ≤ 59.5 Gy N [% from n of grade]	*P* value†
Nausea (56)				0.09
Grade 1	37 [66]	14 [38]	23 [62]	
Grade 2	16 [29]	11 [69]	5 [31]	
Grade 3	3 [5]	1 [33]	2 [66]	
Anorexia (42)				0.87
Grade 1	23 [55]	13 [57]	10 [43]	
Grade 2	17 [40]	8 [47]	9 [53]	
Grade 3	2 [5]	1 [50]	1 [50]	
Vomiting (28)				0.48
Grade 1	25 [89]	12 [48]	13 [52]	
Grade 2	2 [7]	0 [0]	2 [100]	
Grade 3	1 [4]	1 [100]	0 [0]	
Diarrhea (15)				0.77
Grade 1	15 [100]	6 [40]	9 [60]	
Abdominal pain (27)				0.48
Grade 1	25 [93]	12 [48]	13 [52]	
Grade 2	2 [7]	2 [100]	0 [0]	
Fatigue (62)				0.79
Grade 1	41 [66]	18 [44]	23 [56]	
Grade 2	19 [31]	10 [53]	9 [47]	
Grade 3	2 [3]	1 [50]	1 [50]	
Constipation (31)				0.33
Grade 1	27 [87]	12 [44]	15 [56]	
Grade 2	4 [13]	3 [75]	1 [25]	
Dehydration (11)				1
Grade 2	7 [64]	3 [43]	4 [57]	
Grade 3	4 [36]	2 [50]	2 [50]	
Reflux‐like symptoms (7)				0.69
Grade 1	7 [100]	4 [57]	3 [43]	
Skin (12)				0.76
Grade 1	12 [100]	5 [42]	7 [58]	
Fever (5)				0.4
Grade 1	4 [80]	3 [75]	1 [25]	
Grade 2	1 [20]	0 [0]	1 [100]	
Anemia				0.59
Grade 3+^a^	3 [4]	2 [67]	1 [33]	
Other^b^	77 [96]	35 [45]	42 [55]	
Lymphopenia				0.053
Grade 3+^a^	63 [79]	33 [52]	30 [48]	
Other^b^	17 [21]	4 [24]	13 [76]	
Thrombocytopenia				0.68
Grade 2+^a^	6 [8]	2 [33]	4 [67]	
Other^b^	74 [92]	35 [47]	39 [53]	
Ascites	30	15 [50]	15 [50]	0.64
Gastrointestinal bleeding	11	4 [36]	7 [64]	0.53

Acute toxicities were graded according to the Common Terminology Criteria for Adverse Events v4.03.

a No grade 5 toxicities were reported.

b Including normal values.

†Fisher's exact test.

## 4 DISCUSSION

The purpose of this analysis was to evaluate the effect of selective RT dose escalation to portions of the GTV away from bowel in patients with unresectable EHCC. Escalated dose of radiation has been shown to be associated with prolonged survival in unresectable intrahepatic cholangiocarcinoma and unresectable pancreatic ductal adenocarcinoma.^18^, ^19^, ^20^ Despite the acceptable toxicity of higher RT doses in our study, we found that selective escalated RT doses (up to 98 Gy BED) did not significantly benefit patients with unresectable EHCC with regard to increasing OS or FFLP. Unlike intrahepatic cholangiocarcinoma, EHCC is almost always close to bowel, limiting the maximal dose and the dose coverage given to the GTV. Another possible reason why we did not see a positive effect of EDR in our cohort of EHCC is selection bias for EDR in patients with perihilar EHCC (Table 2). Patients with perihilar EHCC are known to have worse prognosis than those with distal EHCC.^21^ Indeed, this difference in prognosis for patients with perihilar and distal EHCC was observed in our results. When calculated from time of diagnosis, OS and FFLP were still not significantly prolonged with EDR (data not shown). Furthermore, when we tried a higher cutoff for defining EDR (BED > 77, n = 18), there was no difference in the patient outcomes between radiation dose groups. Our results demonstrate that local tumor complications represent a major cause of morbidity and mortality for patients with locally advanced, unresectable EHCC, emphasizing the need for more effective multimodality treatments. Furthermore, toward personalized approaches to management, we have identified several prognostic variables that may guide future trial designs.

To our knowledge, this is the largest unresectable EHCC cohort that has been analyzed for the effect of EDR and the cause of death analysis. Ghafoori et al^8^ noticed opposite results in their study where most unresectable EHCC patients treated with RT had metastatic rather than local disease progression. However, their cohort was limited to 37 patients. Median OS was reported to be 10‐16.5 months in studies focusing on RT in unresectable EHCC.^12^, ^14^, ^22^ However, our cohort of patients had higher median OS survival (18.7 months).

Elevated baseline peripheral NLR indicates systemic inflammation and has been reported to be associated with worse prognosis in several types of cancers.^23^ Our data demonstrate that high baseline NLR (>5.3) is associated with shorter FFLP and OS. A recent study has shown similar trends with a slightly lower cutoff (5) for EHCC cases that were resected with a curative intent, but this did not reach statistical significance.^24^ An earlier study has shown a lower NLR cutoff (3) that also predicted survival for both advanced and resected cases of perihilar cholangiocarcinoma.^25^

Our study shows that RT with or without concurrent chemotherapy is well tolerated for the treatment of EHCC. This is consistent with our previously published results about tolerability of upper abdominal RT with concurrent capecitabine.^26^ We observed grade 3+ lymphopenia in patients who received EDR which could be due to chemotherapy or fractionated RT.^27^ Recently, IMRT technique is considered as an alternative to 3D conformal RT in upper abdominal malignancies.^28^ Despite higher radiation doses with IMRT, we observed lower GI toxicities in patients who received treatment with IMRT, compared to 3D. This is consistent with other studies which demonstrated that IMRT use spares normal tissue and is associated with lower GI toxicities in upper abdominal malignancies.^29^, ^30^, ^31^, ^32^ The use of IMRT technique did not compromise local control. Tumor size and distance from nearest GI mucosa were not taken into consideration while analyzing the protective role of IMRT.

There are limitations of this retrospective study which we acknowledge. This single‐institution cohort study naturally is limited by potential confounding factors. There could be potential selection bias in the pretreatment decisions for escalated dose RT, as well as some missing data in the laboratory results and the reported toxicities, due to the retrospective data collection. Also, local progression was reported based on imaging or clinical reporting of local progression which may not meet Response Evaluation Criteria in Solid Tumors version 1.1. The pre‐RT chemotherapy regimens were not standardized in terms of regimen and timing. Despite these limitations, this study is relatively large for this rare tumor type and represents the most comprehensive description of EDR in EHCC.

## 5 CONCLUSION

RT dose escalation to portions of the GTV away from bowel does not appear to benefit patients with unresectable nonmetastatic EHCC. Still, median overall survival in our cohort of patients was better than median OS previously reported in the literature for unresectable EHCC treated with RT. More effective radiation treatment options need to be developed for these patients to achieve higher radiation doses while protecting nearby organs. IMRT is associated with lower rates of acute toxicity compared to 3D techniques. NLR is a readily available indicator of systemic inflammation that may have a role as a prognostic biomarker of EHCC.

## CONFLICT OF INTEREST

The authors have no conflicts to disclose.
