# Supplementary figures and images for: Dose escalation of radiotherapy in unresectable extrahepatic cholangiocarcinoma
Source: Cancer Med. 2018 Aug 27;7(10):4880–92. doi: 10.1002/cam4.1734 (PMC6198206; doi:10.1002/cam4.1734)

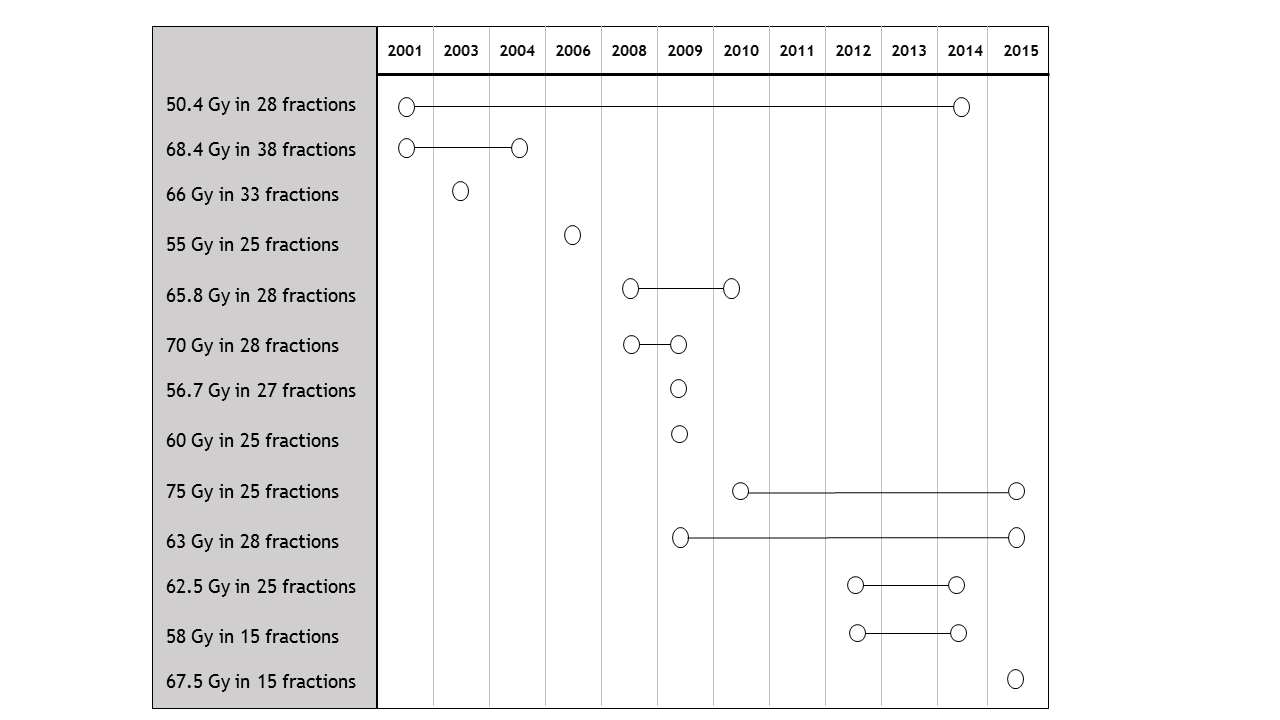

Supplement: Supplementary file 1 [file CAM4-7-4880-s001.tif]

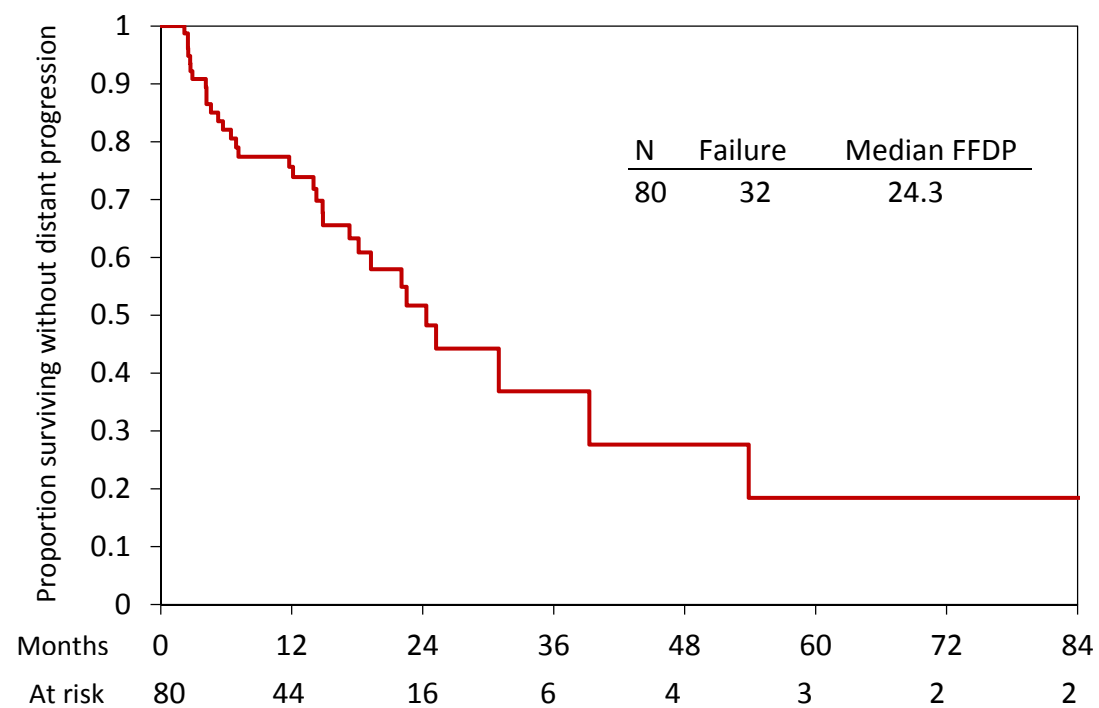

Supplement: Supplementary file 2 [file CAM4-7-4880-s002.pdf]

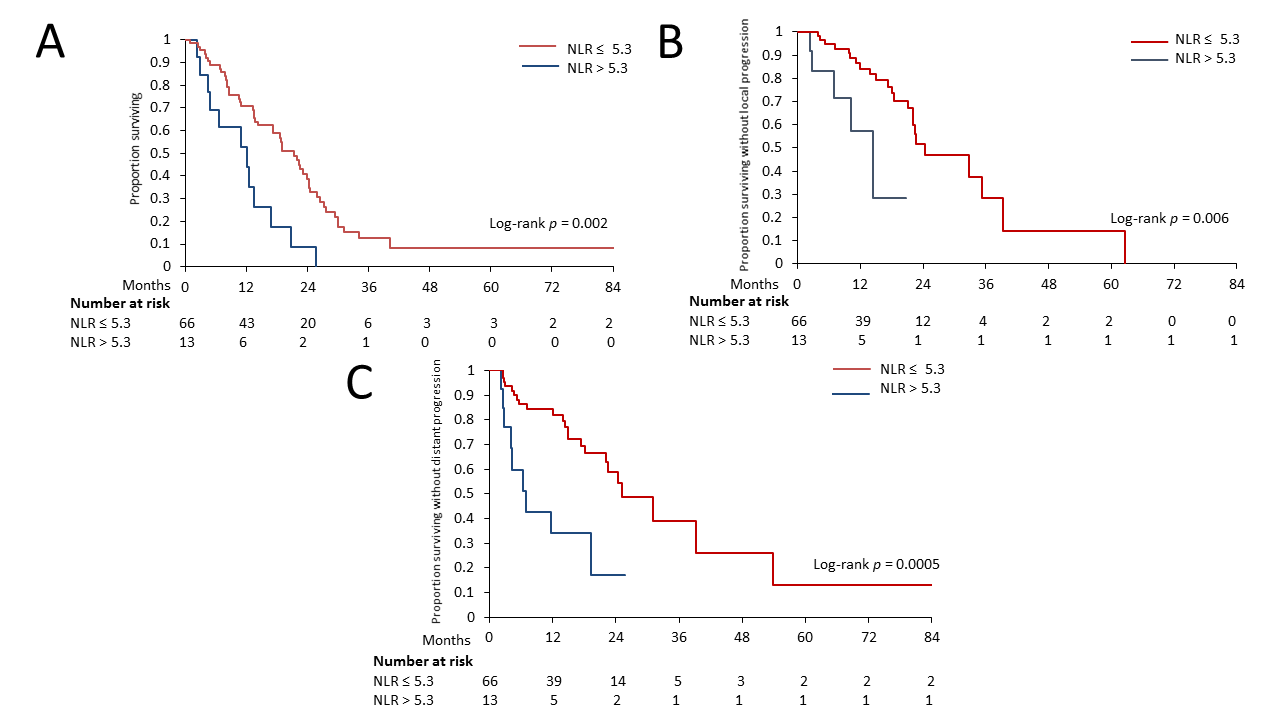

Supplement: Supplementary file 3 [file CAM4-7-4880-s003.tif]
