# Supplementary material for: Dose escalation of radiotherapy in unresectable extrahepatic cholangiocarcinoma
Source: Cancer Med. 2018 Aug 27;7(10):4880–92. doi: 10.1002/cam4.1734 (PMC6198206; doi:10.1002/cam4.1734)
Supplement: Supplementary file 4 [file CAM4-7-4880-s004.docx]

**Supplementary-Table S1:** Univariate survival analysis of overall survival (OS), freedom from local progression (FFLP), and freedom from distant progression (FFDP) for all patients.

|  | **Definition** | **Univariate OS analysis** | | **Univariate FFLP analysis** | | **Univariate FFDP analysis** | |
| --- | --- | --- | --- | --- | --- | --- | --- |
|  |  | *P value* | *HR [95% CI]* | *P value* | *HR [95% CI]* | *P value* | *HR [95% CI]* |
| **Baseline NLR*** |  | **0.04** | 1.09 [1-1.16] | 0.08 | 1.13 [0.98-1.25] | **0.007** | 1.13 [1.03-1.22] |
| **Radiation dose** | High (>50.4 Gy) | 0.4 | 1.23 [0.73-2.05] | 0.4 | 1.4 [0.7-3] | **0.02** | 2.36 [1.17-4.9] |
|  | Standard or low (≤50.4 Gy) | Reference | | Reference | | Reference | |
| **Site of EHCC** | Perihilar | **0.004** | 2.5 [1.3-5.37] | 0.6 | 1.2 [0.53-3.28] | **0.03** | 2.64 [1.08-7.9] |
|  | Distal | Reference | | Reference | | Reference | |
| **Local progression on chemotherapy prior to RT** | Yes | **0.02** | 2.88 [1.17-6.11] | **0.01** | 5.77 [1.57-17.27] | 0.08 | 2.9 [0.85-7.65] |
|  | No | Reference | | Reference | | Reference | |
| **Portal vein involvement** | Yes | **0.04** | 1.89 [1.02-3.4] | 0.1 | 2 [0.8-4.68] | 0.9 | 0.95 [0.37-2.15] |
|  | No | Reference | | Reference | | Reference | |
| **Gross tumor volume (GTV)*** |  | **0.002** | 1.004 [1.001-1.007] | 0.3 | 1.003 [0.99-1.007] | 0.07 | 1.003 [0.99-1.007] |
| **Normalized baseline CA19-9*†** |  | **0.0007** | 1.001 [1.0005-1.002] | 0.1 | 1 [0.999-1.002] | 0.3 | 0.99 [0.99-1.0005] |
| **Age** |  | 0.1 | 1.01 [0.99-1.03] | 0.7 | 1 [0.98-1.04] | 0.9 | 1.001 [0.97-1.03] |
| **Gender** | Male | 0.5 | 1.17 [0.7-1.98] | 0.2 | 0.6 [0.27-1.3] | 0.5 | 0.78 [0.39-1.6] |
|  | Female | Reference | | Reference | | Reference | |
| **Overall stage** | III or IV | **0.002** | 2.77 [1.4-6.11] | 0.09 | 2.2 [0.9-6.8] | 0.2 | 1.74 [0.75-4.79] |
|  | I or II | Reference | | Reference | | Reference | |
| **ECOG performance status** | 2 or 3 | **0.008** | 3.36 [1.4-7.26] | 0.8 | 0.78 [0.04-4] | 0.2 | 2.22 [0.63-6.09] |
|  | 0 or 1 | Reference | | Reference | | Reference | |
| **Use of concurrent chemotherapy** | Yes | 0.13 | 0.5 [0.26-1.2] | 0.58 | 0.7 [0.23-2.99] | **0.03** | 0.3 [0.13-0.88] |
|  | No | Reference | | Reference | | Reference | |

*As a continuous variable.
† Normalized baseline CA19-9 was not included in multivariate analysis due to missing values.
*Abbreviations:* HR = hazards ratio; CI = confidence interval; NLR = neutrophil lymphocyte ratio; RT = radiotherapy; EHCC = extrahepatic cholangiocarcinoma; ECOG = Eastern Cooperative Oncology Group
