# Supplementary material for: Dose escalation of radiotherapy in unresectable extrahepatic cholangiocarcinoma
Source: Cancer Med. 2018 Aug 27;7(10):4880–92. doi: 10.1002/cam4.1734 (PMC6198206; doi:10.1002/cam4.1734)
Supplement: Supplementary file 5 [file CAM4-7-4880-s005.docx]

**Supplementary-Table S2:** Multivariate survival analysis of overall survival (OS), freedom from local progression (FFLP), and freedom from distant progression (FFDP) for all patients using NLR as a categorical variable.

|  | **Definition** | **Multivariate OS analysis** | | **Multivariate FFLP analysis** | | | | **Multivariate FFDP analysis** | |
| --- | --- | --- | --- | --- | --- | --- | --- | --- | --- |
|  |  | *P value* | *HR [95% CI]* | *P value* | | | *HR [95% CI]* | *P value* | *HR [95% CI]* |
| **Baseline NLR (Cutoff = 5.3)** | NLR > 5.3 | 0.38 | 1.4 [0.63-3.1] | **0.04** |  | 3.5 [1.05-10.3] | | **0.01** | 3.7 [1.3-9.78] |
|  | NLR ≤ 5.3 | Reference | | Reference | | | | Reference | |
| **Site of EHCC** | Perihilar | 0.6 | 1.3 [0.46-4] |  | | |  | 0.2 | 2.05 [0.66-7.3] |
|  | Distal | Reference | |  | | | | Reference | |
| **Local progression on chemotherapy prior to RT** | Yes | 0.09 | 2.76 [0.86-5.9] | **0.01** | | | 5.7 [1.5-18.05] | 0.13 | 2.8 [0.68-9.23] |
|  | No | Reference | | Reference | | | | Reference | |
| **Portal vein involvement** | Yes | 0.1 | 1.74 [0.88-3.33] | 0.4 | | | 1.48 [0.56-3.64] |  |  |
|  | No | Reference | | Reference | | | |  | |
| **Gross tumor volume (GTV)*** |  | **0.035** | 1.004 [1-1.007] |  | | |  | 0.98 | 0.97 [0.06-11.1] |
| **Age*** |  | **0.005** | 1.04 [1.01-1.07] |  | | |  |  |  |
| **Gender** | Male |  | | 0.4 | | | 0.7 [0.31-1.6] |  |  |
|  | Female |  | | Reference | | | |  |  |
| **Overall stage** | III or IV | 0.2 | 2.07 [0.69-6.96] | 0.6 | | | 1.3 [0.48-4.2] |  |  |
|  | I or II | Reference | | Reference | | | |  | |
| **ECOG performance status** | 2 or 3 | **0.029** | 2.9 [1.1-7.05] |  | | |  | 0.69 | 1.28 [0.33-4.07] |
|  | 0 or 1 | Reference | |  | | | | Reference | |
| **Radiation dose** | High (>50.4 Gy) |  | |  | | |  | 0.67 | 1.2 [0.5-2.9] |
|  | Standard or low (≤50.4 Gy) |  | |  | | | | Reference | |

*As a continuous variable.
† Normalized baseline CA19-9 was not included in multivariate analysis due to missing values.
*Abbreviations:* HR = hazards ratio; CI = confidence interval; NLR = neutrophil lymphocyte ratio; RT = radiotherapy; EHCC = extrahepatic cholangiocarcinoma; ECOG = Eastern Cooperative Oncology Group.
